# Convalescing: Reflections in a Post-pandemic World

**DOI:** 10.7759/cureus.30620

**Published:** 2022-10-23

**Authors:** Quratulanne H Jan, Lindsay K Sonstein, Sunil K Sahai

**Affiliations:** 1 Family Medicine, University of Texas Medical Branch at Galveston, Galveston, USA; 2 Internal Medicine, University of Texas Medical Branch at Galveston, Galveston, USA

**Keywords:** primary care, professional communication, communication, technology, staffing, collaborative medicine, covid-19 retro

## Abstract

The COVID-19 pandemic brought with it many hardships to the world as a whole. Mass infection and casualties due to disease state were rampant, which affected many families. Lockdown drove up the incidence of depression and isolated people from their loved ones. The toll on the general population was high, as was the toll on the medical community, a subset of the general population. It was a time of death and devastation, with supply chain issues creating personal protective equipment shortages and staffing being affected by illness, fear, age, and expertise. Despite the constraints, many places were able to rally staff together to provide adequate staffing for healthcare delivery purposes to serve our communities. Cross-specialty collaboration in the outpatient and inpatient setting as well as the use of technology aided our service to our community to help persevere through the many surges of the pandemic and come out with lessons learned as well as reflections.

## Editorial

The COVID-19 pandemic brought with it many hardships to the world and has affected humankind on a scale not previously seen. Mass infection and casualties due to disease state were rampant, which affected many families and communities. Lockdown drove up the incidence of depression and isolated people from their loved ones. The toll on the general population was high, as was the toll on the medical community tasked with providing needed medical care. It was a time of death and devastation, with supply chain issues creating personal protective equipment shortages and staffing being affected by illness, fear, age, and expertise. Despite the constraints, many hospitals and medical centers were able to rally together to provide adequate staffing for healthcare delivery purposes to serve our communities. Cross-specialty collaboration in the outpatient and inpatient setting and the use of technology aided our service to our community to help persevere through the many surges of the pandemic and come out with lessons learned as well as reflections.

Practicing medicine during an international pandemic brought with it many unprecedented challenges, one of which was staffing. The Galveston Island-based University of Texas Medical Branch (UTMB) has a robust disaster preparedness infrastructure in place given the frequency of hurricanes on the Gulf Coast. The unprecedented nature of the pandemic posed new challenges. Hurricanes are predictable, with clear stages from formation over the ocean, to landfall, to post-storm recovery. The pandemic did not have clearly defined phases and raised the level of anxiety due to our inability to forecast the future. Our institution initially went on lockdown around the time most did in the country, in the early spring of 2020. Out of this situation and movement toward delivering telehealth services, many plans were put in place for backup teams ranging from general Internal Medicine (IM) and its specialties, surgical specialties, and Family Medicine (FM). In theory, if needed, efforts were made to provide help by requesting “all hands on deck.” Borne out of this necessity were clinics strategically placed in different geographical areas to serve our community and patients. Being a large institution, with community-based care and academic practices, all areas were affected.

On the academic practice side, we developed the COVID clinic model, which utilized a mixture of Family Medicine and Internal Medicine house staff and faculty to service the clinic seven days a week, 8-12 hours a day. This created an effective way to test and triage patients, allowing us to optimize our admission process to prioritize those who met the shifting criteria for admission. The jointly staffed COVID clinic model also gave us the opportunity to collaborate among different departments and administrative units of the health system. It allowed us to start breaking down the different silos of care that exist and move toward the institutional goal of comprehensive and compassionate patient care for all.

With this collaboration came the challenge of communicating with everyone to ensure those involved in the care of patients with SARS-CoV-2 were on the same page. This took the shape of several email chains, the development of a WhatsApp group for inpatient COVID-19 care, and a weekly call with inpatient faculty with representatives from Family Medicine, Infectious Disease, and Internal Medicine. For rapid communication, WhatsApp was chosen due to its end-to-end encrypting, making it easier for us to share important yet sensitive information with our colleagues [1,2].

While the population sheltered in place, the Division of General Medicine was able to expand dedicated COVID inpatient teams and accommodate the needs of the patients by redeploying faculty and residents from closed clinics to the hospital. Unique among academic medical centers, UTMB has an attached Texas Department of Criminal Justice (TDCJ) Hospital serving the needs of patients who are incarcerated. Inpatient medical care at TDCJ Hospital Galveston is overseen by the faculty and residents of the Internal Medicine residency program. As IM teams were being deployed to cover the surge of patients in the TDCJ Hospital, the surge was also affecting our main teaching hospital, Jennie Sealy Hospital, putting a strain on staffing. A new system of delivery was developed to provide coverage for the surge in COVID patients in Jennie Sealy. Inspired by the success of the outpatient COVID clinic, we created a team that was staffed with Internal Medicine and Family Medicine residents and respective specialty faculty on alternating weeks. This allowed faculty and residents in Internal Medicine to balance service and education better, take some much-needed time off, and further improve the collaboration between both departments.

With widespread vaccination and as COVID-19 becomes endemic, the complexity of care and mortality has become predictable, and surges do not threaten to overwhelm healthcare workers as they once did. The world has opened up and has shifted back to the pre-pandemic way of life; we have learned that a collaborative model is the way forward [3,4]. Collaborating between specialties has allowed us to serve our community in a complete manner by allowing us to expand and offer services in pandemic times, for the good of humanity. It has also allowed us to provide adequate staffing by relying on multiple specialties of residents and faculty with training that is appropriate to deal with the illness as present and allowed educational and clinical experience to our trainees. COVID will become endemic and become part of the repertoire of circulating seasonal respiratory infections. A collaborative approach also helps promote work-life balance as adequate primary care staffing improves the risk of burnout and incorporating technology allows for efficiencies [1,2]. The pandemic forced our two primary care specialties and training programs to work in a collaborative fashion in both the outpatient and inpatient settings. We see much promise in this model to address future pandemics and other situations where staffing and resources are strained.
